# Removal of Emerging Pollutants from Water Using Environmentally Friendly Processes: Photocatalysts Preparation, Characterization, Intermediates Identification and Toxicity Assessment

**DOI:** 10.3390/nano11010215

**Published:** 2021-01-15

**Authors:** Nina Finčur, Paula Sfîrloagă, Predrag Putnik, Vesna Despotović, Marina Lazarević, Maria Uzelac, Biljana Abramović, Paulina Vlazan, Cătălin Ianăși, Tünde Alapi, Máté Náfrádi, Ivana Maksimović, Marina Putnik-Delić, Daniela Šojić Merkulov

**Affiliations:** 1 Department of Chemistry, Biochemistry and Environmental Protection, University of Novi Sad Faculty of Sciences, Trg Dositeja Obradovića 3, 21000 Novi Sad, Serbia; nina.fincur@dh.uns.ac.rs (N.F.); vesna.despotovic@dh.uns.ac.rs (V.D.); marina.lazarevic@dh.uns.ac.rs (M.L.); maria@dh.uns.ac.rs (M.U.); biljana.abramovic@dh.uns.ac.rs (B.A.); 2 National Institute of Research and Development for Electrochemistry and Condensed Matter, Dr. Aurel Păunescu Podeanu 144, 300569 Timişoara, Romania; paulasfirloaga@gmail.com (P.S.); vlazanp@yahoo.com (P.V.); 3 Department of Food Technology, University North, Trg dr. Žarka Dolinara 1, 48000 Koprivnica, Croatia; 4 Coriolan Dragulescu “Institute of Chemistry”, 24 Mihai Viteazu Bvd., 300223 Timisoara, Romania; cianasic@yahoo.com; 5 Department of Inorganic and Analytical Chemistry, University of Szeged, Dóm tér 7, 6720 Szeged, Hungary; alapi@chem.u-szeged.hu (T.A.); nafradim@chem.u-szeged.hu (M.N.); 6 Faculty of Agriculture, University of Novi Sad, Trg Dositeja Obradovića 8, 21000 Novi Sad, Serbia; ivanam@polj.uns.ac.rs (I.M.); putnikdelic@polj.uns.ac.rs (M.P.-D.)

**Keywords:** sol–gel synthesis, TiO_2_, ZnO, MgO, removal efficiency, antibiotic, herbicide, toxicity, reaction intermediate

## Abstract

Pharmaceuticals and pesticides are emerging contaminants problematic in the aquatic environment because of their adverse effects on aquatic life and humans. In order to remove them from water, photocatalysis is one of the most modern technologies to be used. First, newly synthesized photocatalysts were successfully prepared using a sol–gel method and characterized by different techniques (XRD, FTIR, UV/Vis, BET and SEM/EDX). The photocatalytic properties of TiO_2_, ZnO and MgO nanoparticles were examined according to their removal from water for two antibiotics (ciprofloxacin and ceftriaxone) and two herbicides (tembotrione and fluroxypyr) exposed to UV/simulated sunlight (SS). TiO_2_ proved to be the most efficient nanopowder under UV and SS. Addition of (NH_4_)_2_S_2_O_8_ led to the faster removal of both antibiotics and herbicide fluroxypyr. The main intermediates were separated and identified for the herbicides and antibiotic ciprofloxacin. Finally, the toxicity of each emerging pollutant mixture and formed intermediates was assessed on wheat germination and biomass production.

## 1. Introduction

All living organisms and various human activities depend on the sources of pure water. Emerging pollutants reach vital aquatic compartments, such as surface water, groundwater and drinking water, at concentrations between ng and µg/L, which have negative influence on the water quality [1]. They originate from pharmaceuticals, pesticides, (recreational) drug abuse, personal care industries, industrial compounds, steroid hormones, etc., naturally or from anthropogenic sources [2] and include domestic, hospital and industrial effluents; runoffs from agriculture, livestock and aquaculture; and landfill leachates. Mentioned sources of pollutants might follow numerous additional pathways [3]; for instance, antibiotics are a group of pharmaceuticals that have been widely used for almost one hundred years for the treatment of infections. The majority of antibiotics cannot be completely metabolized or biodegraded by humans; hence, their deposits in the environment pose threats to health and ecology by inducing the development of antibiotic-resistant bacteria and affecting the metabolisms of living organisms. Therefore, great attention has been paid to studying consequences of antibiotics and their abuse in society [4].

Ciprofloxacin (CIP, l-cyclopropyl-6-fluoro-4-oxo-7-(piperazin-l-yl)-1,4-dihydroquinoline-3-carboxylic acid) belongs to fluoroquinolones, the most important class of synthetic antibiotics, which has been widely used for a broad spectrum of antimicrobial activities. The ubiquitous presence of CIP has been reported in the effluent of municipal wastewaters treatment plants, river basins, soil and sediments from river basin [5]. The above-mentioned presence of CIP in the environments will accelerate the appearance of antibiotic-resistant bacteria that will consequently at high concentrations cause damages to the immunity [6].

Ceftriaxone disodium salt hemi(heptahydrate) (CEF, (6R,7R)-7-{[(2Z)-2-(2-amino-1,3-thiazol-4-yl)-2-methoxyiminoacetyl]amino}-3-[(2-methyl-5,6-dioxo-1H-1,2,4-triazin-3-yl)sulfanylmethyl]-8-oxo-5-thia-1-azabicyclo[4.2.0]oct-2-ene-2-carboxylic acid) is a third-generation cephalosporin antibiotic, which has a broad-spectrum activity against Gram-positive bacteria and expanded Gram-negative ones [7]. Although a solution of CEF is reported to be unstable [8,9,10], CEF was found in natural [11] and waste waters [12,13]. It has been reported that cephalosporin intermediates can be more toxic and persistent than the parent drugs [14].

The frequent occurrence of pesticides in wastewater and associated environmental hazards has also heightened concerns over public health due to their high toxicity and bio-recalcitrant nature. As mentioned before, these contaminants are continuously released into the aquatic environment through various anthropogenic activities [15].

Tembotrione (TEM, 2-(2-chloro-4-methylsulfonyl-3-(2,2,2-trifluoroethoxy)methyl)benzoyl cyclohexane-1,3-dione) is the most recently commercialized triketone herbicide worldwide in 2007 and is used as a post-emergence herbicide on all maize varieties [16]. TEM is classified in category I as very toxic to aquatic organisms (microalgae). For instance, TEM and formulated TEM showed the strongest impact on *Pseudokirchneriella subcapitata* microalgae [17,18]. Biodegradation and ozonation with different chemicals (flurtamone, fluopyram, TEM, flufenacet, fluoxetine, sitagliptine and 4:2 fluorotelomer sulfonate) proved that there are sources and pathways of trifluoroacetate, whose elevated concentrations (>100 μg/L) were found in a major German rivers [19].

Fluroxypyr (FLU, (4-amino-3,5-dichloro-6-fluoro-2-pyridinyl) oxyacetic acid) is a selective post-emergent systemic herbicide widely used in agriculture. It was introduced in Europe for post-emergence control of annual and perennial broad-leaf weeds in small grains such as wheat, barley, oats and croplands [20,21]. FLU is classified by EPA as Toxicity Category II and as “not likely” a human carcinogen. However, subchronic toxicity assays in rats showed nephrotoxicity, increased kidney weight, hispathological lesions and decreased renal function [22]. The large input of FLU into the farmlands has led to its widespread occurrence in ecosystem including soils, lakes and even underground water [23]. Major properties of investigated pollutants are summarized in Table 1.

Bearing in mind all of the above, there is a need to develop an efficient, safe and environmentally friendly process to remove emerging pollutants from water systems. Depending on physicochemical properties of the pollutant and on the type of process used, the efficiency of conventional wastewater treatment plants varies. The main mechanisms for removal of emerging pollutants occurring during the secondary treatment at wastewater treatment plants are biological and/or chemical transformation and sorption [1]. Advanced oxidation processes (AOPs) include the production of highly reactive oxidizing species, such as hydroxyl radicals, and these processes are able to unselectively degrade different pollutants [24]. One of the AOPs, heterogeneous photocatalysis, is based on the irradiation with appropriate intensity of wide-band-gap semiconductors, which in that way generate electrons and holes and consequential chain reactions producing hydroxyl radicals [25]. Presently, different materials, such as TiO_2_, ZnO, SnO_2_, WO_3_, ZnS and MgO, etc. are used as semiconductors in the processes of heterogeneous photocatalysis [26]. As alternatives to H_2_O_2_-based advanced oxidation processes, whereby H_2_O_2_ dissociates to form the hydroxyl radical, persulfate activation processes involving the formation of the highly reactive sulfate radical (${\mathrm{SO}}_{4}^{•-}$) have attracted increasing attention because of the reduction potential of ${\mathrm{SO}}_{4}^{•-}$ (*E*^0^(${\mathrm{SO}}_{4}^{•-}$/${\mathrm{SO}}_{4}^{2-}$) = 2.5–3.1 V_NHE_ compared to that of ^•^OH; *E*^0^(^•^OH/OH^−^) = 1.8–2.7 V_NHE_. This indicates that the strong oxidizing power of ${\mathrm{SO}}_{4}^{•-}$ enables the persulfate activation system to oxidatively treat a broad spectrum of aquatic pollutants [27]. In addition, persulfate as an electron acceptor has been utilized in environmental remediation systems.

In this work, nanopowders (TiO_2_, ZnO and MgO) were synthesized using the sol–gel method and characterized by X-ray diffraction (XRD) analysis, FTIR spectrometry, UV/Vis spectrophotometry, BET and SEM/EDX measurements. Their efficiency was investigated for the removal of selected emerging pollutants, two antibiotics (CIP and CEF) and two herbicides (TEM and FLU) under UV and simulated sunlight (SS). Moreover, addition of (NH_4_)_2_S_2_O_8_ on the efficiency of pollutants removal was studied. In addition, toxicity of starting compound and its intermediates formed during the removal process on wheat germination, seedling growth, photosynthetic pigments and concentration of malondialdehyde (MDA) were investigated. Reaction intermediates that formed during the removal process were studied in detail.

## 2. Materials and Methods

### 2.1. Synthesis of TiO_2_, ZnO and MgO Nanopowders

Titanium(IV) isopropoxide (C_12_H_28_O_4_Ti), magnesium nitrate (Mg(NO_3_)_2_ × 6H_2_O) and zinc nitrate hexahydrate (Zn(NO_3_)_2_ × 6H_2_O) were used as precursors to synthesize titanium dioxide, zinc oxide and magnesium oxide. All the reagents were of high purity and were purchased from Sigma-Aldrich. Titanium dioxide, zinc oxide, and magnesium oxide were prepared by means of the sol–gel method. The studied materials were synthesized as follows: for all samples, a mixture (50 mL) of water–ethylene glycol (1:1) was used as solvent. To synthesize titanium dioxide, 2 mL of titanium isopropoxide was added to the above solution with continuous stirring. The result was an opalescent solution, which was stirred for 2 h at room temperature. In the case of magnesium oxide synthesis, 2.56 g of Mg(NO_3_)_2_ × 6H_2_O was added to the solvent mixture. The obtained solution was stirred with a magnetic stirrer for 30 min, and then 2 M NaOH solution was added until pH = 9. In the case of zinc oxide synthesis, 2.97 g of (Zn(NO_3_)_2_ × 6H_2_O) was added to the solvent mixture. The obtained solution was stirred on the magnetic stirrer for 30 min, and then 2 M NaOH solution was added until pH = 9. For all the syntheses performed, the temperature was raised to 120 °C and maintained until the solvent evaporated. The obtained powders were heat-treated at 500 °C for 2 h.

### 2.2. Materials Characterization

The XRD analysis was performed using a X-ray diffractometer (PANalyticalX’Pert) with Cu-Kα radiation (λ = 1.5406 Å) at 40 kV and 30 mA. FTIR spectrometry was performed using a JASCO FT/IR-4200 spectrometer, in KBr pellets, in the 500–4000 cm^−1^ range. The absorption spectra were recorded with a LAMBDA 950 UV/Vis/NIR spectrophotometer. BET surface areas were measured by nitrogen adsorption at 77 K on a Nova1200e apparatus. Surface morphology (SEM/EDX) was investigated by scanning electron microscopy, Inspect S, FEI Company.

### 2.3. Measurements of Photocatalytic Activity

The efficiencies of TiO_2_, ZnO, and MgO nanopowders were evaluated by the removal of aqueous solution of two antibiotics, CIP (≥98%, Sigma-Aldrich) and CEF (≥99%, Sigma-Aldrich), as well as two herbicides, TEM (99.4%, Sigma-Aldrich) and FLU (99%, Fluka). The aqueous solutions were made using ultrapure water. All stock solutions were protected from light and stored at room temperature, with the exception of CEF, which was stored at 4 ± 1 °C. Removal experiments were performed as previously described by our group [31]. Irradiation under UV was performed using 125 W high-pressure mercury lamp (Philips, HPL-N, emission bands in the UV region at 304, 314, 335 and 366 nm, with maximum emission at 366 nm and intensity of 2.6 × 10^−3^ W/cm^2^ in the visible region and 1.4 × 10^–2^ W/cm^2^ in UV region) together with an appropriate concave mirror. On the other hand, irradiation under SS was carried out using a 50 W halogen lamp (Philips) with the intensity of 0.1 W/cm^2^ in the visible region and 2.2 × 10^–4^ W/cm^2^ in the UV region. The suspension was thermostatted at 25.0 °C in the stream of O_2_ (3.0 mL/min) and then irradiated. During irradiation, the suspension was stirred at a constant rate under the continuous O_2_-flow. Experiments were performed using 20 mL of emerging pollutant solution, where their initial concentration in all experiments was 0.05 mM, while catalyst loading was 0.5 mg/mL. The obtained suspensions were filtered through Millipore (Millex-GV, MA, USA, 0.22 μm) membrane filters. The preliminary check confirmed the absence of pollutants adsorption on the filters.

In the experiments where effects of the (NH_4_)_2_S_2_O_8_ were studied (Merck, Skopje, North Macedonia), this compound was added in two concentrations of 0.125 and 0.5 mM in the reaction suspension. This obtained suspension was stirred in the dark before irradiation for achieving adsorption–desorption equilibrium. Initially, the blank experiment was performed to display that no reaction took place in the absence of UV/SS irradiation after 15 min of stirring in the dark. Kinetic studies of all investigated pollutants were monitored by ultra-fast liquid chromatography with diode array detector (UFLC−DAD) using Shimadzu UFLC-DAD, equipped with an Eclipse XDB-C18 column (150 mm × 4.6 mm i.d., particle size 5 μm, 25 °C). All chromatograms were recorded in the wavelength range of 190–300 nm. Mixtures of acetonitrile (ACN, 99.9%, Sigma-Aldrich, St. Louis, MO, USA) and water were used as mobile phase for analysis of all studied pollutants, which was acidified with mass fraction of 0.1% H_3_PO_4_ (85%, Sigma-Aldrich, St. Louis, MO, USA). Conditions in which the analysis was performed for CIP had UV/vis DAD detector at 279 nm (wavelength of CIP maximum absorption). The ratio of mobile phase for ACN and water was 20:80 (*v*/*v*), and flow rate was 0.8 mL/min. In the case of CEF, the UV/vis DAD detector was set to 260 nm (wavelength of maximum absorption of CEF), and the ACN and water ratio was 15:85 (*v*/*v*) with flow rate of 1.0 mL/min. Conditions for TEM analysis using UFLC−DAD had UV/vis DAD detector at 284 nm (wavelength of TEM maximum absorption). The mixture of mobile phase, ACN and water, was 60:40 (*v*/*v*), and flow rate was 1.0 mL/min. For the FLU, UV/Vis DAD detector was set at 212 nm (wavelength of FLU maximum absorption), and the mobile phase with flow rate of 1.0 mL/min was a mixture of ACN and water in ratio 50:50 (*v*/*v*).

Delta Ohm HD 2102.2 (Padova, Italy) was used for the UV energy fluxes measurements. The radiometer was fitted with the LP 471 UVA sensor (spectral range 315–400 nm), and in the case of visible energy, the radiometer was fitted with the LP 471 RAD (spectral range 400–1050 nm).

For LC–MS evaluation of intermediates, selected suspensions of investigated pollutants (0.1 mM) were irradiated in different time intervals under UV/SS und analyzed using an Agilent 1100 HPLC coupled with an LC/MSD VL mass spectrometer equipped with Electrospray Ionization (ESI) source and a triple quadrupole analyzer (QqQ). The used column was a Kinetex 2.6 µm XB-C18 100 A (pore size 2.6 µm). The mobile phase consisted of ACN (VWR, HPLC grade) and aqueous solution of 0.1% formic acid (VWR; 99–100%), their ratio (*v*/*v*) was 15:85 for both CIP and CEF; 30:70 for the FLU, and 50:50 for TEM. The flow rate was set to 0.60 mL/min. When measuring in positive ion mode, 4000 V capillary and 50 V fragmentation voltages were used; in the negative mode, these values were set to 3000 V and 40 V, respectively.

### 2.4. Germination and Toxicity Measurements

For the toxicity assessments, selected suspensions of each pollutant (0.05 mM) were irradiated after 15 min of adsorption by UV-light (t = 60 min) and by SS (t = 180 min), then filtered and analyzed.

Experiments were done to compare the effects of antibiotics (CIP and CEF) and herbicides (TEM and FLU) and the efficiency of treatments (irradiation with catalyst) in alleviation of their toxicity of on initial growth phases of wheat. Wheat seeds were germinated in the presence of solutions of: (1) intact CIP, CEF, TEM and FLU (these treatments were followed by number 1), filtered suspensions of (2) CIP, CEF, TEM and FLU treated by SS during 180 min in the presence of catalyst (these treatments were followed by number 2) or filtered suspensions of (3) CIP, CEF, TEM and FLU irradiated by UV light during 60 min in the presence of catalysts (these treatments were followed by number 3). After germination, seedlings were grown on a complete ½ strength solution after Hoagland and Arnon [32], without any further addition of analyzed substances.

For the germination tests, 30 seeds of wheat, cultivar NS40S, were placed on a filter paper in a Petri dish (*R* = 9 cm) with the addition of 7.5 mL of tested solutions or deionized water (controls) and covered with a lid. Petri dishes were placed in the incubator at 26 °C in the dark in 3 replications. Germinated seeds were counted after 24, 48 and 72 h. Three days after sowing (72 h) 5 seedlings from each Petri-dish (15 per treatment) were used to assess fresh weight (FW) of roots and shoots. The remaining seedlings were transplanted in plastic pots (*V* = 750 mL) filled with ½ strength Hoagland solution. Sixteen plants were placed per pot in 3 replications. Pots were placed in a growth chamber (RK-340 CH, Kambič) where they were grown under the following conditions: 12 h day/night period, 23 °C/19 °C temperature regime, 45% humidity, 80% ventilation, light provided by FLUORA 18W/77 lamps. After 8 and 13 days in the chambers, when plants were 11 and 16 days old, respectively, FW of their roots and shoots were assessed. At d11, besides the FW, concentration of photosynthetic pigments and MDA (a measure of the integrity of cell membranes) were assessed, in three independent replications. The concentrations of photosynthetic pigments were measured in acetone extracts of freshly harvested leaves using molar extinction coefficients according to Holm [33] and von Wettstein [34]. The concentrations of MDA were assessed as described by Devasagayam et al. [35]. Statistical analyses were done by the TIBCO Statistica v.13 software. Analyses of variance and LSD tests were performed at *p* ≤ 0.05.

## 3. Results and Discussion

### 3.1. Structural Characteristics

**Figure 1:**
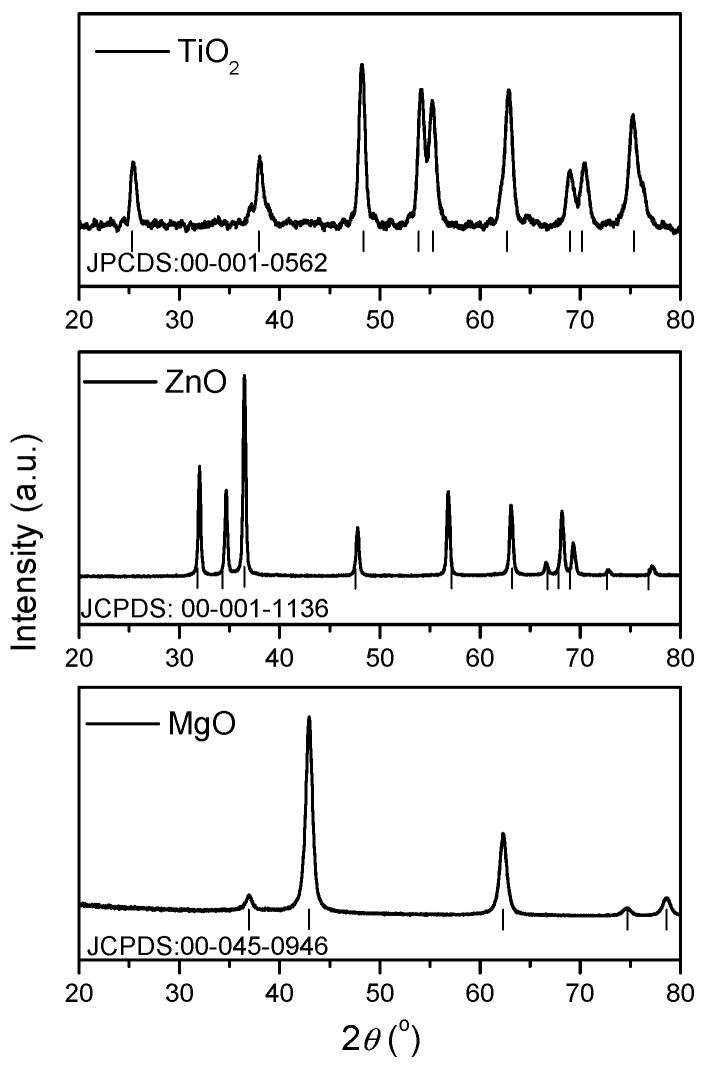
XRD patterns for TiO_2_, ZnO and MgO synthesized through the sol–gel method.

The crystal structures of the TiO_2_, ZnO and MgO powders were studied using XRD patterns. Crystallographic data were obtained by Rietveld refinement using the PANalytical X’Pert HighScore Plus program. In Figure 1 are shown XRD results for the three materials, and analysis of spectra showed that all of the diffraction peaks can be indexed as a single phase.

Thus, it can be seen that the sample TiO_2_ showed characteristic peaks of anatase as an only phase, with lattice parameters *a* = *b* = 3.7300 Å and *c* = 9.3700 Å, space group *I*4/*amd* according to JCPDS card no. 00-001-0562. In the case of ZnO, this crystallized in typical hexagonal phase with wurtzite structure, space group *P*63*mc* and lattice parameters: *a* = *b* = 3.25229 Å and *c* = 5.2096 Å according to JCPDS card no. 00-001-1136. The XRD pattern of the MgO sample indicated a unique phase without any other crystalline impurities detected. The diffraction peaks corresponded to the cubic crystalline phase with space group *Fm*-3*m* and lattice parameters *a* = *b* = *c* = 4.2194 Å in compliance with JCPDS card no. 00-045-0946.

**Figure 2:**
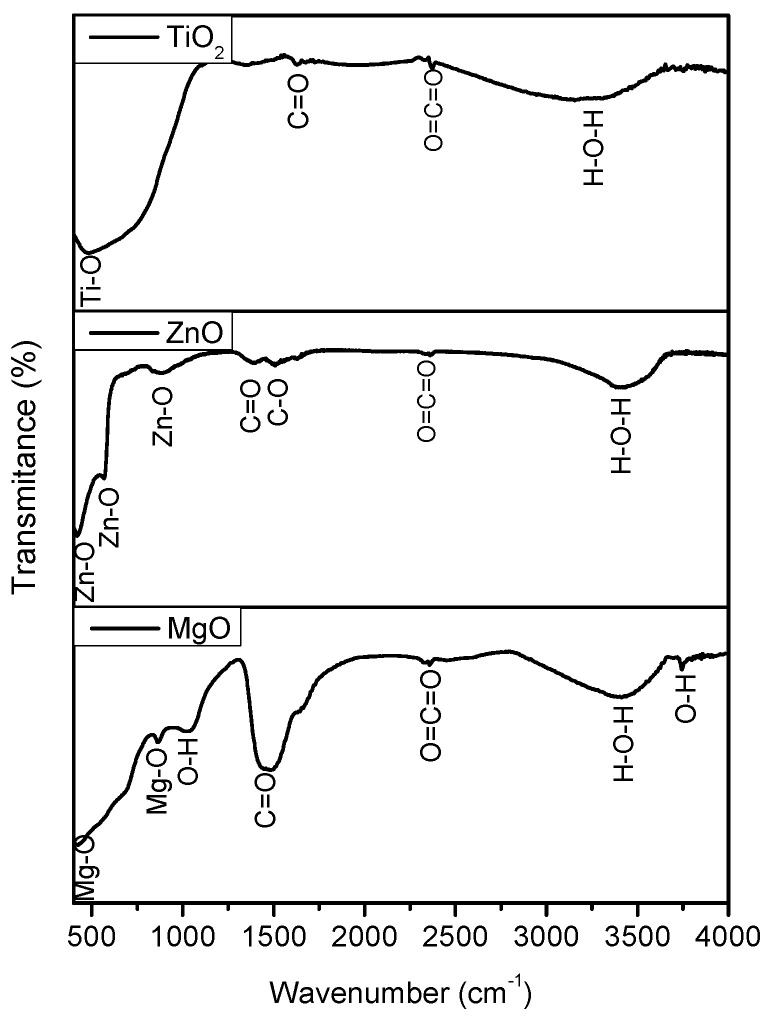
The transmission FTIR spectra of TiO_2_, ZnO, and MgO synthesized through sol-gel method.

FTIR spectra of TiO_2_, ZnO and MgO nanomaterials can be observed in Figure 2, where it shows a series of absorption bands in the range of 400–4000 cm^–1^. The spectra presented common bands such as the broad band in the field of 3200–3500 cm^–1^ due to the O–H stretching vibration of the absorbed water molecule and surface hydroxyl group [36]. In addition, a low-intensity band was found in the area 2350–2370 cm^–1^; the absorption is because of the presence of CO_2_ molecules in the air [37].

The intense peak at 1468.7 cm^−1^ for MgO was associated with vibrational mode of H−ion bonded to Mg^2+^ on different co-ordination sites [38]. There were other insignificant bands at 880 cm^−1^, 1039.7 cm^−1^ and in the range of 1380–1630 cm^−1^ in the spectra. These absorption bands were likely related to CO_2_ absorbed from the air atmosphere. The FTIR spectra showed that the bands from interval 429–880 cm^–1^ can be attributed to the Me-O bond. These bands are attributed to the Me-O bonds with different modes stretching and vibration modes [39].

**Figure 3:**
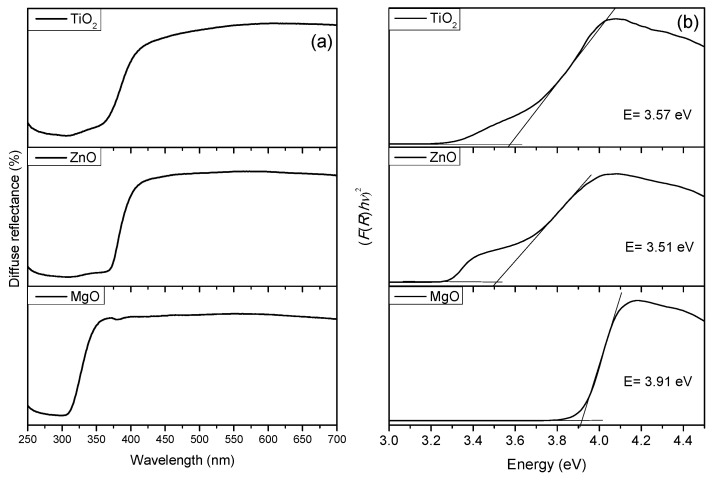
UV–Vis spectra (**a**) and band gap energies (**b**) of studied materials.

The UV-Vis diffuse reflectance spectra of TiO_2_, ZnO and MgO samples are shown in Figure 3a. The band gap energy for powdered samples were calculated from their diffuse reflectance spectra, using of the Kubelka–Munk method. The band gap estimated for oxide samples (Figure 3b) in this paper are as follows: TiO_2_-*E*_g_ = 3.57 eV, ZnO-*E*_g_ = 3.51 eV and MgO-*E*_g_ = 3.91 eV.

**Figure 4:**
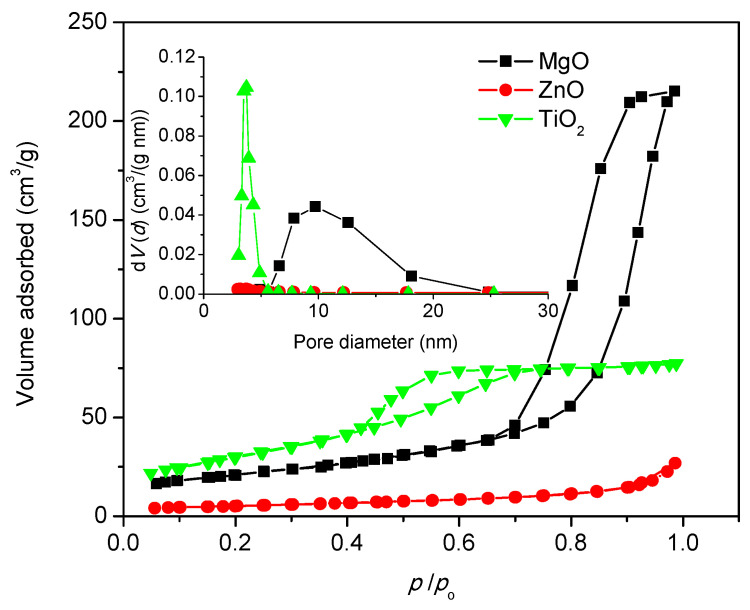
Adsorption–desorption isotherms for TiO_2_, ZnO and MgO materials.

The materials were degassed for 5 h at T = 100 °C with Nova1200e apparatus and analyzed at 77 K with nitrogen. The BET method was used for evaluating the surface area, and BJH was used for pore size distribution. The total pore volume was obtained from the last point of adsorption branch from hysteresis near 1 *P*/*P*_0_. Adsorption–desorption isotherm obtained after performing the analysis was presented in Figure 4. In the inset of Figure 4, the average distribution of pores was shown. TiO_2_ isotherm is of type IVa with hysteresis of type H2b, according to IUPAC classification [40]. In the case of a Type IVa isotherm, capillary condensation was accompanied by hysteresis. This occurs when the pore width exceeds a certain critical width, which is dependent of the adsorption system and temperature [41,42,43].

The MgO material is a type IVa isotherm with hysteresis of type H1. Type H1 hysteresis has also been found in networks of inkbottle pores where the width of the neck size distribution is similar to the width of the pore/cavity size distribution. The textural properties obtained from isotherms were presented in Table 2.

**Figure 5:**
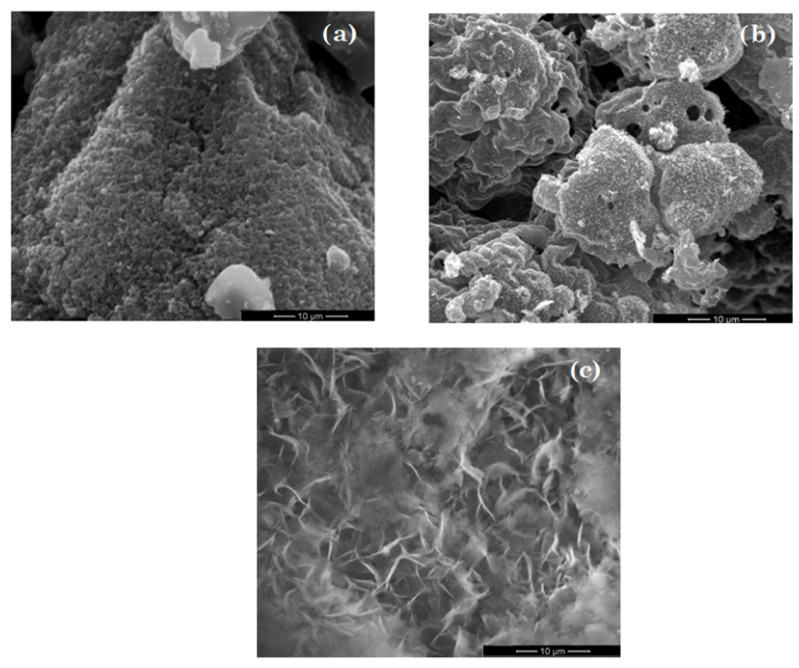
SEM images of TiO_2_ (**a**), ZnO (**b**) and MgO (**c**).

Figure 5 shows surface morphology of TiO_2_ (a), ZnO (b) and MgO (c) materials obtained through sol–gel method. As can be seen, both TiO_2_ and ZnO were strongly agglomerated in asymmetrical formations. In the case of materials based on MgO, it can be seen that the morphology of a micrometer-sized aggregates consisted of irregular rod and piece.

**Figure 6:**
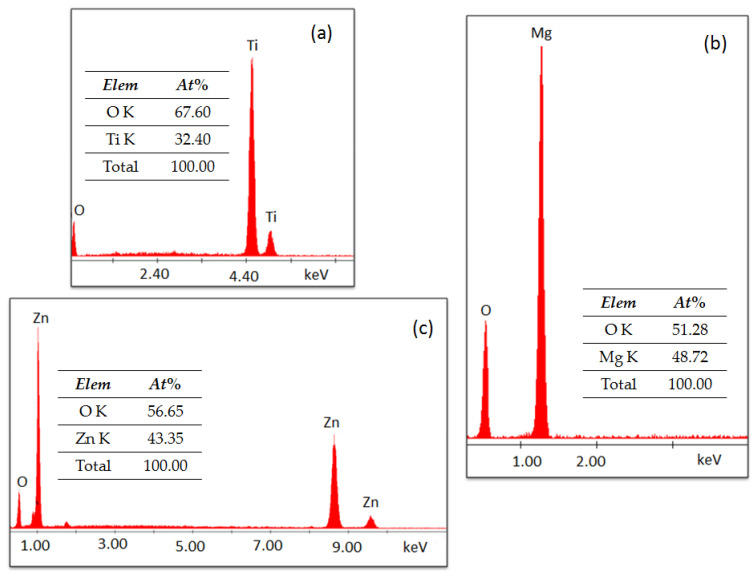
EDX spectra for TiO_2_ (**a**), MgO (**b**) and ZnO (**c**).

Furthermore, to confirm the phase purity and elemental composition of studied materials, EDX analysis was carried out. The obtained results confirm the high phase purity and presence of Ti and O elements, in the case of TiO_2_, Zn and O in the case of ZnO and Mg and O for MgO, as shown in Figure 6.

### 3.2. Removal Efficiency

Efficiencies of newly synthesized nanopowders (TiO_2_, ZnO and MgO) were investigated in the removal of selected emerging pollutants, two antibiotics and two herbicides under UV and SS. In order to improve removal of pollutants, (NH_4_)_2_S_2_O_8_ was added in two concentrations (0.125 and 0.5 mM) to the reaction mixtures, consisting of emerging pollutant and the catalyst, which showed the highest removal efficiency. As mentioned, in the presence of (NH_4_)_2_S_2_O_8_ it can be expected increased removal of pollutants because of the photochemical activation of (NH_4_)_2_S_2_O_8_, resulting in sulfate radical anions [44].

**Figure 7:**
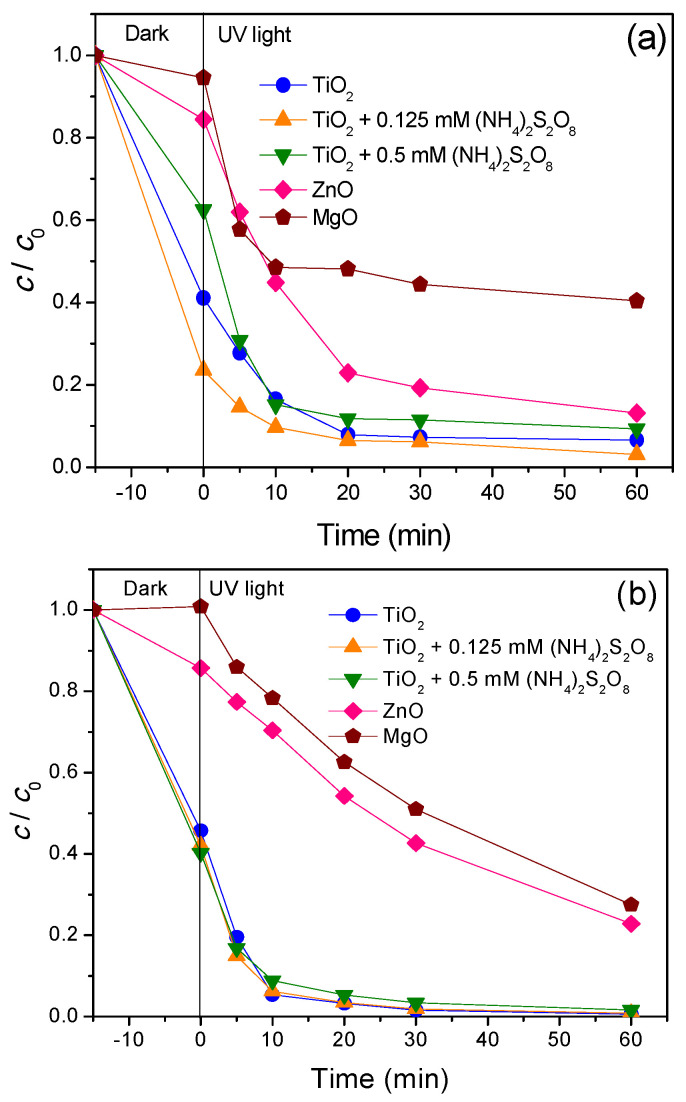
Removal of antibiotics (**a**) CIP and (**b**) CEF (0.05 mM) from water using different catalysts (0.5 mg/mL) in the presence/absence of (NH_4_)_2_S_2_O_8_ under UV light.

Removal kinetics of antibiotics (CIP and CEF) under UV light was presented in Figure 7. Figure 7a shows that among newly synthesized nanopowders, TiO_2_ had the highest efficiency for CIP removal where after 75 min, 93.4% of substrate was removed. Slightly less activity was shown by ZnO, where 86.9% of CIP was removed, and the lowest activity had MgO (59.6%).

As mentioned before, addition of (NH_4_)_2_S_2_O_8_ on the CIP removal efficiency with TiO_2_ was investigated. As can be seen from Figure 7a, the highest CIP removal was obtained in the presence of 0.125 mM (NH_4_)_2_S_2_O_8_. Interestingly, increased concentration of 0.5 mM (NH_4_)_2_S_2_O_8_ slightly reduced process efficiency. Negative effect of the (NH_4_)_2_S_2_O_8_ presence can be explained as concentrations above the optimal value inducing the scavenging of sulphate radicals by (NH_4_)_2_S_2_O_8_ or through the self-quench of sulphate radicals [44].

In the case of CEF, obtained results under UV light were presented on Figure 7b. They showed that TiO_2_ again was the most efficient nanopowder where CEF was completely removed from the aqueous solution after 75 min. In the case of ZnO and MgO efficiency, removal of CEF was 77.2% and 72.5%, respectively, after 75 min of process. Since TiO_2_ proved to be the most efficient nanopowder using UV light, this catalyst was used in further examination with (NH_4_)_2_S_2_O_8_. As can be seen, the addition of (NH_4_)_2_S_2_O_8_ is not conducive to CEF removal. Namely, the CEF removal remains unchanged regardless of the addition of persulfate because all active sites on the catalyst are occupied by the adsorbed CEF and persulfate reacts only with photogenerated ^•^OH in the solution. Similar results were obtained in our previous studies [45].

**Figure 8:**
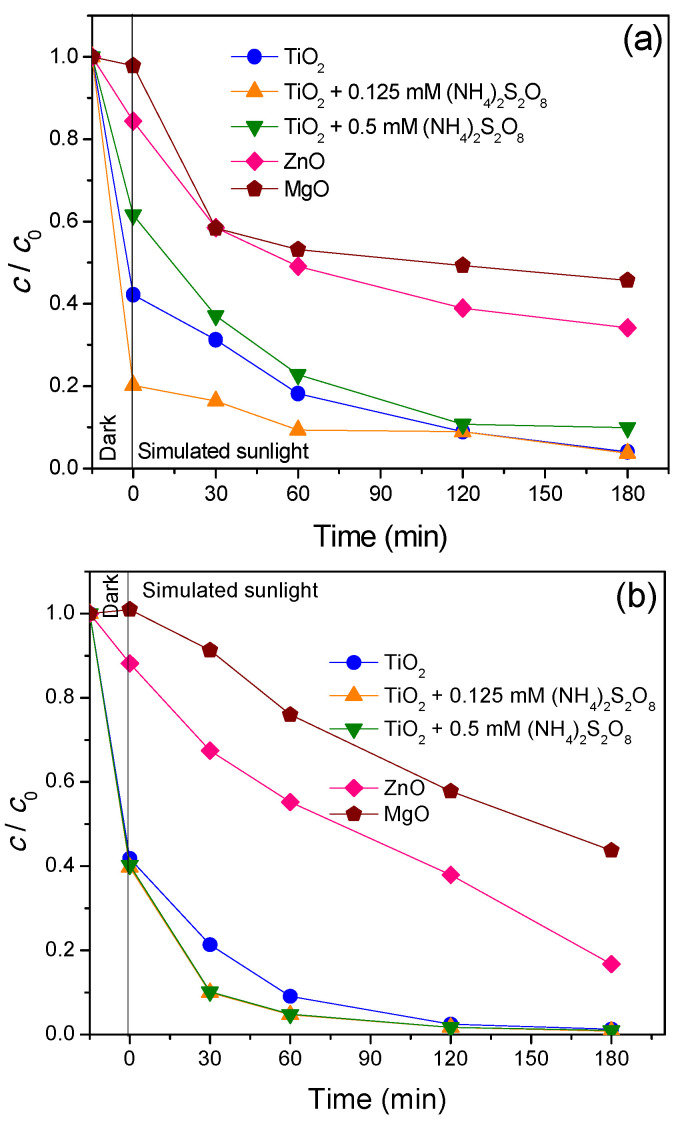
Removal of antibiotics (**a**) CIP and (**b**) CEF (0.05 mM) from water using different catalysts (0.5 mg/mL) in the presence/absence of (NH_4_)_2_S_2_O_8_ under SS.

In order to examine the influences of the radiation types, all aforementioned nanopowders were investigated for the removal of two antibiotics using SS radiation (Figure 8). As can be seen, the removal of CIP and CEF was somewhat slower using SS. For CIP removal (Figure 8a), TiO_2_ showed the highest efficiency, where after 195 min, roughly 96% was removed. Under the same length of time, lower removal efficiency was obtained with ZnO (65%) and MgO (54%). Addition of (NH_4_)_2_S_2_O_8_ to the reaction suspension of CIP and TiO_2_ showed that its lower concentration increased the removal of CIP during initial 135 min, and after that, the removal remained the same as with the absence of (NH_4_)_2_S_2_O_8_. Similar to before, higher concentration of (NH_4_)_2_S_2_O_8_ slightly decreased CIP removal.

Newly synthesized TiO_2_, ZnO and MgO were examined for the removal of CEF under SS (Figure 8b). Again, TiO_2_ outperformed all examined nanopowders and fully removed CEF after 195 min. ZnO and MgO removals of CEF were 83.3% and 56.3%, respectively for the same process time. Since TiO_2_ proved to be the most efficient nanopowder, it was used for further investigations with (NH_4_)_2_S_2_O_8_. Different concentrations of (NH_4_)_2_S_2_O_8_ were added to the suspension of CEF/TiO_2_, and the CEF removal was faster during the first 60 min of irradiation, while after 180 min, this remained the same as in the system with sole TiO_2_. This implies that hydroxyl radicals had a major role in photocatalytic oxidation of the CEF.

Results presented in Figure 7 and Figure 8 showed significant adsorption of CIP and CEF on the TiO_2_ surface (~60% of CIP and CEF adsorbed). Using (NH_4_)_2_S_2_O_8_ had no effect on the adsorption of CEF, but CIP showed significantly higher adsorption in the presence of 0.125 mM of (NH_4_)_2_S_2_O_8_ (around 75%). For the case of ZnO, adsorptions of both antibiotics were around 15%, while for MgO they were below 10% with adsorption of CEF that was not even registered.

**Figure 9:**
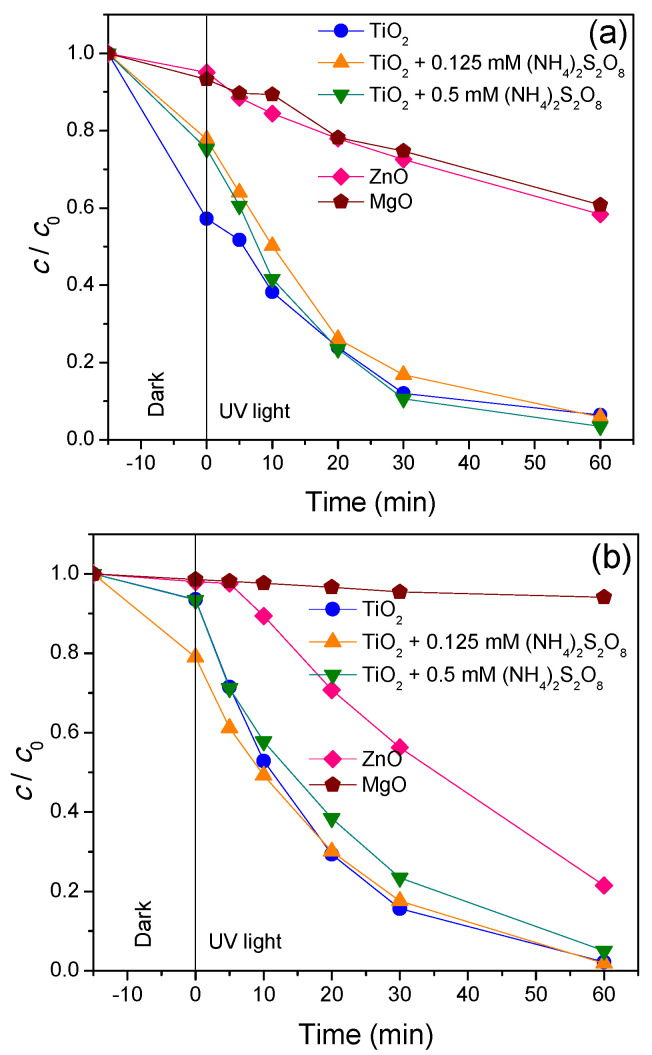
Removal of herbicides (**a**) TEM and (**b**) FLU (0.05 mM) from water using different catalysts (0.5 mg/mL) in the presence/absence of (NH_4_)_2_S_2_O_8_ under UV light.

**Figure 10:**
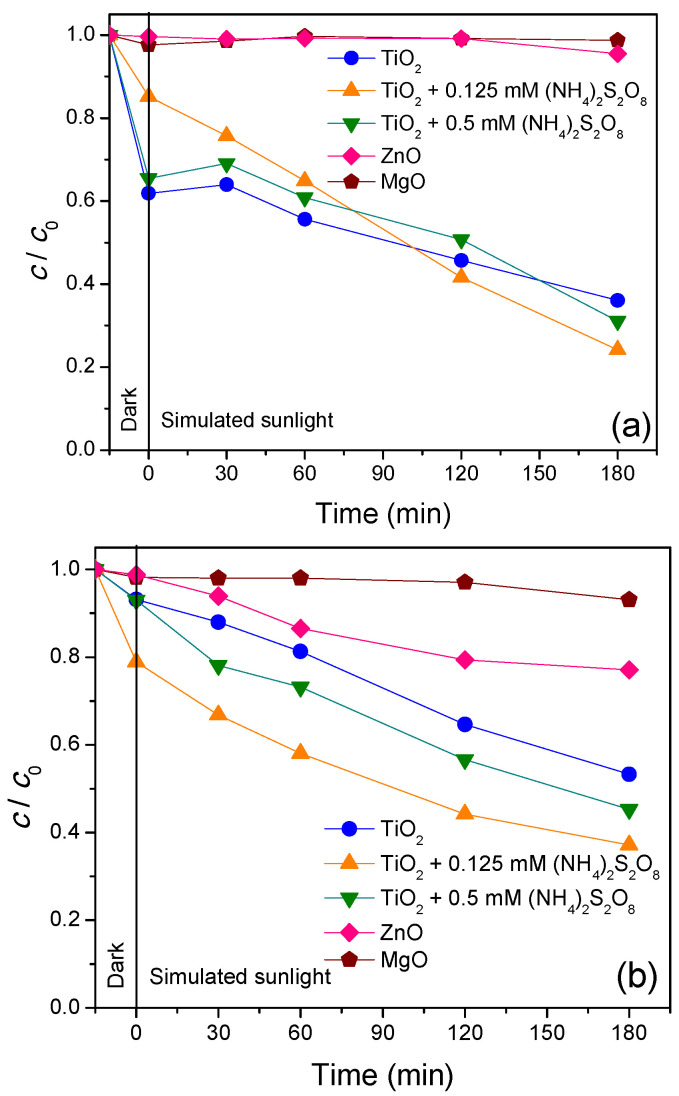
Removal of herbicides (**a**) TEM and (**b**) FLU (0.05 mM) from water using different catalysts (0.5 mg/mL) in the presence/absence of (NH_4_)_2_S_2_O_8_ under SS.

Kinetics of removal efficiency of selected herbicides (TEM and FLU) using UV and SS were presented in Figure 9 and Figure 10. Figure 9a shows the efficiency of TEM removal by UV, where TiO_2_ removed ~95% of TEM after 75 min. After the same time frame, addition of (NH_4_)_2_S_2_O_8_ had no effect of TEM removal regardless of the added concentrations. Under the same conditions (for 75 min), ZnO catalyst removed 42% of TEM, while MgO removed only 39% (Figure 9a). Further, TiO_2_ removed 97% of FLU from the aquatic suspension during the same time (Figure 9b). Addition of (NH_4_)_2_S_2_O_8_ into the UV/TiO_2_ system, had no influence on the final FLU removal efficiency. Lastly, 79% of the FLU was removed by ZnO, while only 6% was removed with MgO during the same time (75 min).

On the other hand, under SS radiation, TiO_2_ removed TEM with lower efficiency of 64% during longer process time of 195 min (Figure 10a). Addition of 0.125 mM (NH_4_)_2_S_2_O_8_ led to a slightly higher removal of TEM (76%), while concentration of 0.5 mM (NH_4_)_2_S_2_O_8_ practically had no effect (Figure 10a). The removal activity of ZnO and MgO over 195 min under SS showed that these nanopowders were inactive regarding the removal of TEM (Figure 10a). Herbicide FLU results exhibited that the highest removal had TiO_2_ where efficiency was 47% after 195 min of process (Figure 10b), while activity of ZnO was lower than TiO_2_ (23%). Substrate removal was practically nonexistent with MgO. The addition of (NH_4_)_2_S_2_O_8_ increased removal activity for TiO_2_ nanopowder, where both concentrations of (NH_4_)_2_S_2_O_8_ increased the removal activity of TiO_2_ at 16% and 8%, respectively. Based on the all obtained results, it can be seen that, as in the case of antibiotics, the herbicides removal efficiency were somewhat slower using SS.

Adsorption studies in the dark for TEM showed that the highest retention was on the surface of TiO_2_, where after 15 min roughly 43% of TEM was adsorbed (Figure 9a and Figure 10a). Interestingly, the presence of (NH_4_)_2_S_2_O_8_ reduced TEM adsorption on the surface of TiO_2_, while adsorptions were unremarkable at 10% for ZnO and MgO. Furthermore, the adsorption of FLU was the highest with TiO_2_ and 0.125 mM of (NH_4_)_2_S_2_O_8_ (Figure 9b and Figure 10b). Here 21% of herbicide was adsorbed for 15 min stirring in the dark. Further addition of (NH_4_)_2_S_2_O_8_ (0.5 mM) had little help for adsorption as it was similar to the sole TiO_2_ system, where after 15 min in the dark, about 7% of herbicide was adsorbed. Adsorption of FLU was completely subsided in the presence of MgO and ZnO catalysts.

In summary, TiO_2_ proved to be the most efficient nanopowder under both UV and SS radiations, and additions of lower concentrations of (NH_4_)_2_S_2_O_8_ facilitated faster removals of both antibiotics and herbicide FLU. Hence, system TiO_2_/(NH_4_)_2_S_2_O_8_ (0.125 mM) was selected for further detailed examinations of degradation intermediates of antibiotics and toxicity assessments. In the case of herbicide TEM, for the above-mentioned experiments, system TiO_2_/(NH_4_)_2_S_2_O_8_ (0.5 mM) under UV light was selected, whereas for SS radiation, system TiO_2_/(NH_4_)_2_S_2_O_8_ (0.125 mM) was chosen.

### 3.3. Impact on Wheat Germination and Growth

Accumulation of antibiotics in crops, among diverse other substances, can be a path of their introduction into the food chain [46,47]. Therefore, the presence of antibiotics and herbicides and intermediates of their decomposition may, on one hand, influence growth of crops and on the other their composition and quality.

#### 3.3.1. Germination

**Figure 11:**
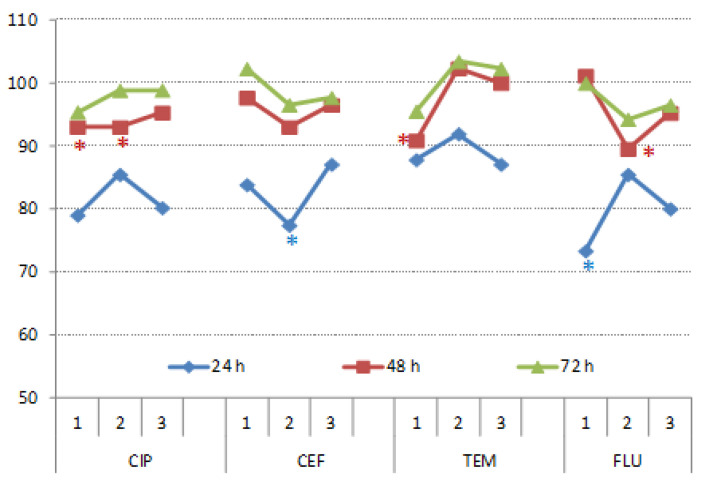
Germination of wheat under CIP, CEF, TEM and FLU treatments recorded after 24, 48 and 72 h. The results were expressed relatively to respective controls (control = 100%). Values with asterisks are significantly different from respective controls.

Wheat germination recorded after 72 h in the presence of CIP, CEF, TEM and FLU was not significantly affected. Nevertheless, those substances initially slowed down germination (after 24 h), especially untreated FLU (Figure 11).

#### 3.3.2. Plant Growth

**Figure 12:**
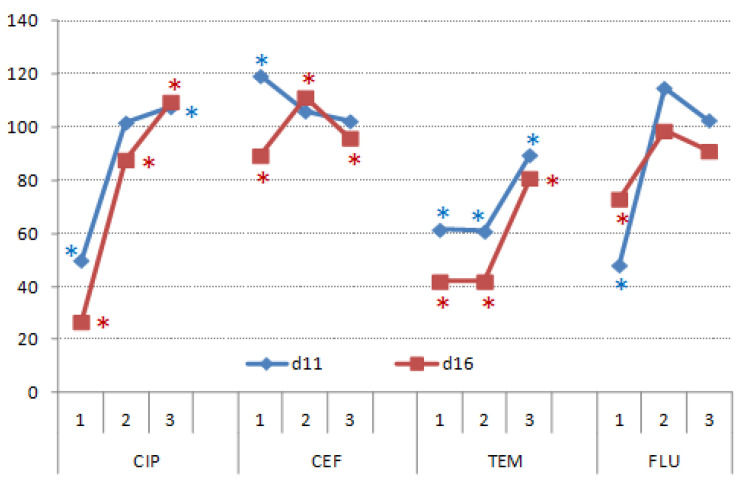
Shoots’ fresh weight of wheat germinated under CIP, CEF, TEM, and FLU treatments and subsequently grown on complete nutrient solution, recorded in 11- and 16-day-old plants. The results are expressed as relative percentages to respective controls (control = 100%). Asterisks indicate significant difference from respective controls.

Shoots’ growth was severely impaired by CIP1, TEM1 and FLU1 but not by CEF (Figure 12). Treatments with SS, UV light and catalyst significantly alleviated retardation of CIP and FLU (CIP2, CIP3 and FLU2, FLU3) on shoots’ biomass. On the contrary, SS and catalyst had no significant effects on shoots’ growth compared to untreated TEM samples. UV light and catalyst significantly reduced toxicity of TEM, but shoots’ biomass was still 20% lower with respect to the control. Overall, CEF3 had the least effect on roots’ growth in comparison to the other CEF treatments, but also all CIP, TEM and FLU treatments.

**Figure 13:**
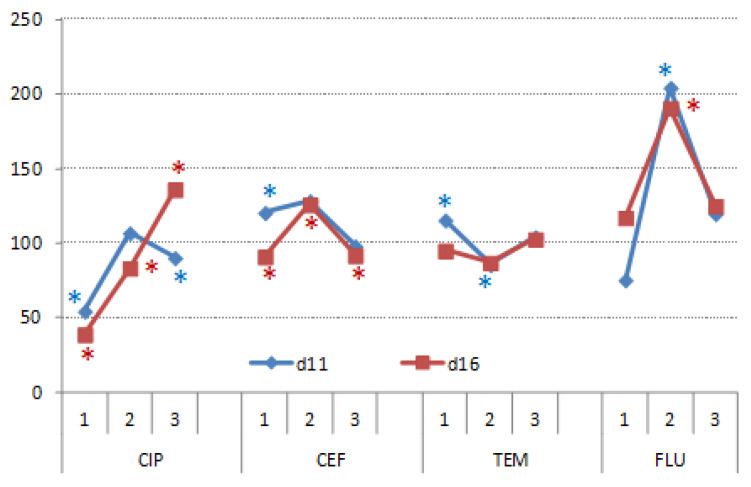
Root fresh weight of wheat germinated under CIP, CEF, TEM and FLU treatments and subsequently grown on complete nutrient solution, recorded in 11- and 16-day-old plants. The results are expressed relatively to respective controls (control = 100%). Asterisks mark values that were significantly different from respective controls.

Roots’ growth was significantly impaired by CIP1 and CIP3 at d11, whereas at d16, differences between roots’ biomass (RMs) were significant between all treatments (Figure 13). CIP1 and CIP2 significantly reduced RMs but CIP3 significantly increased it by nearly 40%. CEF1 significantly increased RMs in plants at d11, but at d16 CEF1 and CEF3, both significantly decreased RMs with respect to the control, whereas CEF2 had significantly higher RMs. TEM affected RMs the least with respect to the other treatments. At d16, differences were not statistically significant, while FLU2 significantly increased RMs, whereas FLU1 and FLU3 did not affect it significantly.

It was found that antibiotics used in veterinary medicine (penicillin, sulfadiazine and tetracycline) affect plant germination and subsequent growth [48], and their effect depends both on plant species and the antibiotic properties. However, lower biomass allocation is found to be common plant response. This was consistent with the literature as aquatic macrophyte *Eichhornia crassipes* under hydroponic conditions exhibited decline in chlorophyll content and alteration of the activity of antioxidant enzymes in the presence of CIP [49].

#### 3.3.3. Photosynthetic Pigments

**Figure 14:**
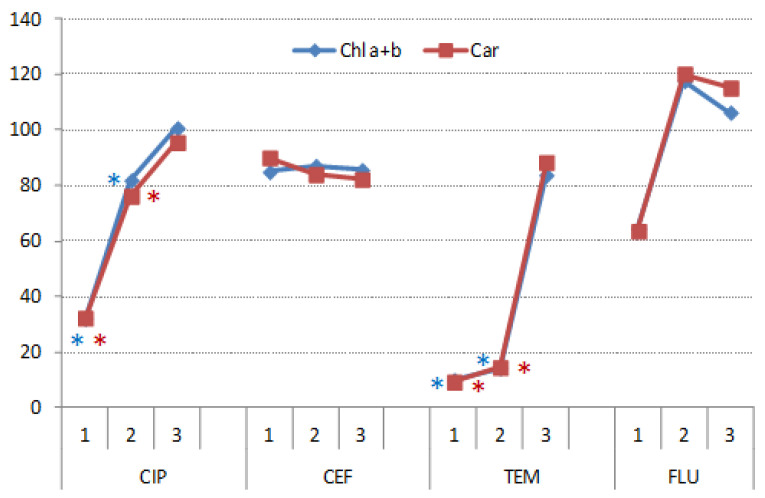
Concentration of photosynthetic pigments (chlorophylls a and b and carotenoids) in the leaves of wheat germinated under CIP, CEF, TEM and FLU treatments and subsequently grown on complete nutrient solution, recorded in 11-day-old plants. The results are expressed relative to respective controls (control = 100%). Asterisks mark values were significantly different from respective controls.

Antibiotic CIP significantly reduced concentration of both chlorophylls and carotenoids (Figure 14). It was previously shown that aquatic macrophyte *Eichhornia crassipes* under hydroponic conditions exhibited decline in chlorophyll content and alteration of the activity of antioxidant enzymes in the presence of CIP [49]. However, treatment with UV light and catalyst alleviated these negative effects. A very similar effect on photosynthetic pigments exhibited TEM, which is not surprising because this herbicide is used to control grassy weeds. Here as well, treatment with UV light and catalyst efficiently removed this inhibition. CEF and FLU did not significantly affect concentrations of photosynthetic pigments, even though FLU1 reduced it by nearly 40%. The fact that FLU is a herbicide used to control broad-leaved weeds and not grassy weeds like TEM can explain the difference in their effects on growth and photosynthetic pigments in wheat [50].

#### 3.3.4. Concentration of Malondyaldehide in Roots and Shoots of Wheat

**Figure 15:**
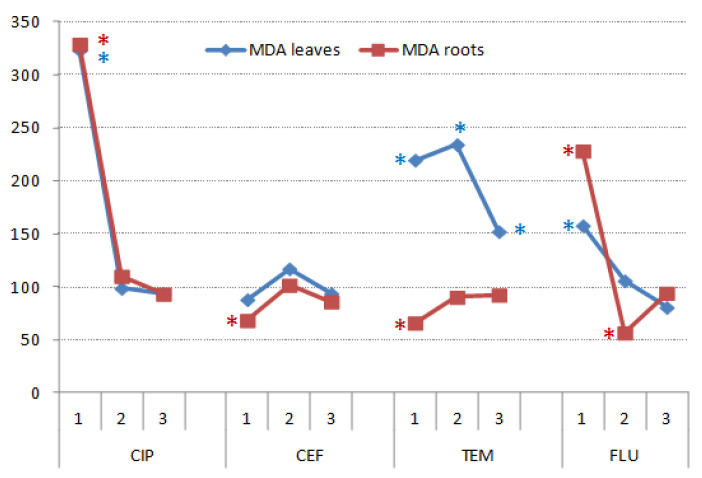
Concentration of MDA in the leaves and roots of wheat germinated under CIP, CEF, TEM and FLU treatments and subsequently grown on complete nutrient solution, recorded in 11-day-old plants. The results are expressed relative to respective controls (control = 100%). Asterisks mark values that were significantly different from respective controls.

Increase in lipid peroxidation of biomembranes, in response to various kinds of stressors, was reflected through changes in concentrations of MDA in plant tissues [51]. In wheat leaves and roots, untreated CIP and FLU strongly increased concentration of MDA in both, leaves and roots (Figure 15). All three TEM treatments significantly increased MDA concentration in leaves. Untreated CEF, TEM and FLU treated by SS and catalyst significantly reduced concentration of MDA in the roots. Except for TEM in leaves, UV and catalyst treatments efficiently brought MDA levels to those of the controls in the leaves and roots alike. Those changes in MDA concentration, associated with different kinds of abiotic stress, including salt, are strongly linked to the overall effectiveness of wheat antioxidant systems [52].

Taken altogether, the results indicated that SS, and especially UV light, with catalyst suppressed very negative effects that CIP exhibited on wheat biomass accumulation, concentration of photosynthetic pigments and integrity of cell membranes. The overall effects of CEF on wheat were much less pronounced than those of CIP and effects of treatments by sunlight; however, UV light and catalyst were somewhat less striking, but still efficient. TEM strongly retarded wheat growth (especially shoots), synthesis of photosynthetic pigments, and strong increase of MDA in the leaves. These adverse effects of TEM were only partially suppressed with treatment of UV light and catalyst, since shoots’ biomass remained significantly lower and MDA concentration significantly higher with respect to the controls. Untreated FLU significantly reduced shoots’ fresh weight and increased MDA content in both shoots and roots. However, photosynthetic pigments were not significantly affected, while treatments of FLU by SS, UV light and catalyst alleviated these adverse effects. Treatment of FLU by UV light and catalyst even resulted in significant increase in root biomass, thus changing the shoot-to-root ratios. The exposure of wheat seeds only during the germination (imbibition) to the tested substances (CIP, CEF, TEM, FLU and their intermediates) allowed assessment of their effects on further growth of young plants and to rate the efficiency of SS and UV light together with catalyst on the changes of the biological effects of untreated CIP, CEF, TEM and FLU.

### 3.4. LC/MS Identification of Pollutant Degradation Intermediates

**Figure 16:**
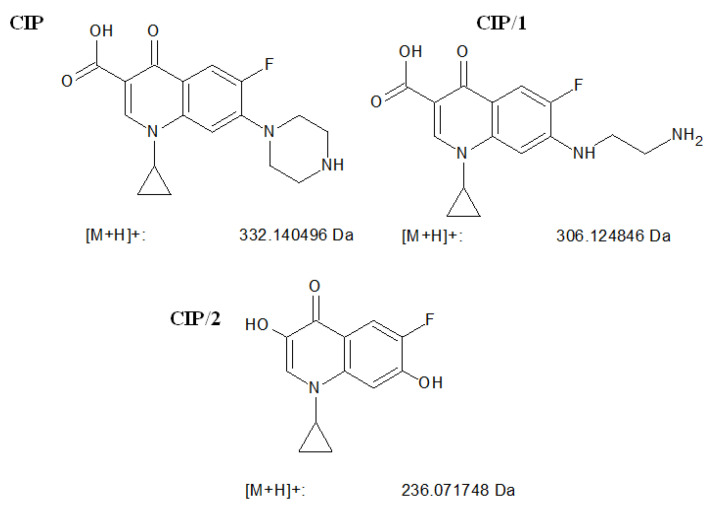
The structure of CIP, and the suggested structure and calculated masses of the formed ions of the detected stable products.

In the case of CIP, identification of the formed intermediates was performed in positive ion mode. The positive ion formed from CIP (*m*/*z* = 332.1) was detected, and after 60 min of irradiation, two stable products could be identified CIP/1 (*m*/*z* = 306.1), and CIP/2 (*m*/*z* = 362.2) (Figure 16). CIP/1 has also been detected by Yu et al. [53] in the case of photocatalytic transformation, and they attributed this formation to the direct attack of valence band holes on CIP adsorbed on the surface of the catalyst. In the case of reactions with hydroxyl radicals, the decarboxylation and hydroxylation of the aromatic ring would be preferred, but such products could not be detected. CIP/2 is suspected to be a significantly degraded product of CIP.

In the case of CEF, measurements were performed both in the positive and negative mode, and although several transformation products were detected, no exact reaction mechanism could be proposed due to the high adsorption and reaction rate of CEF on the catalyst surface. The instability of the solutions was also found to be significant, despite its reported relative stability [9]. The higher amounts of transformation products could be attributed to the reaction of CEF with persulfate.

**Figure 17:**
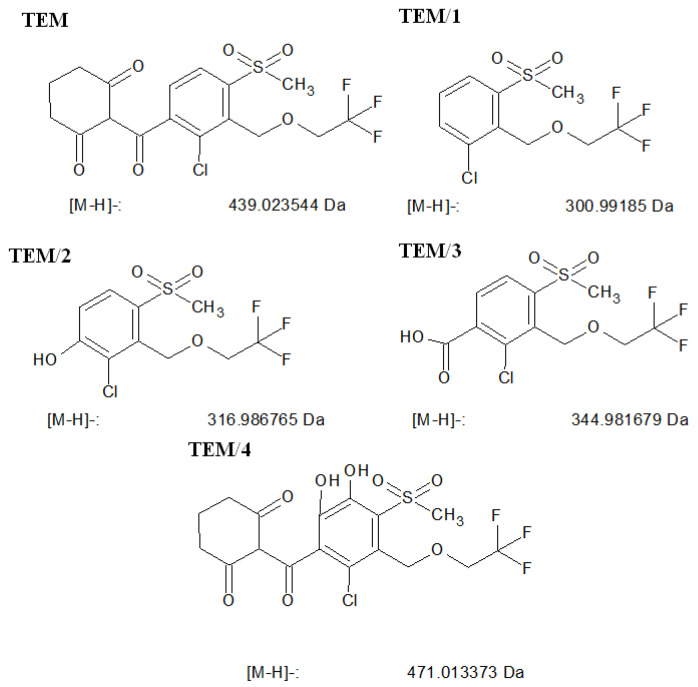
The structure of TEM, and the suggested structure and calculated masses of the formed ions of the detected stable products.

In the case of TEM, the measurements were performed in negative ion mode. TEM (*m*/*z* = 439.2) and four stable products were identified: TEM/1 (*m*/*z* = 301.1), TEM/2 (*m*/*z* = 317.1), TEM/3 (*m*/*z* = 345.1) and TEM/4 (*m*/*z* = 471.1) (Figure 17). TEM/4 is the dihidroxylated product of TEM, and the formations of such products were generally attributed to the attack of hydroxyl radicals on the aromatic ring. TEM/3 was formed from the cleavage of the cyclohexanedione ring, while TEM/2 is the product of the decarboxylation of TEM/3 followed by hydroxylation. Both products have been reported also to form as the result of chlorination [18] and ozonation [54] of tembotrione-containing water. TEM/1 is suspected to form from the dehydroxylation of TEM/2 product. According to these results, the transformation of TEM and its intermediates can be mainly attributed to hydroxyl radicals.

**Figure 18:**
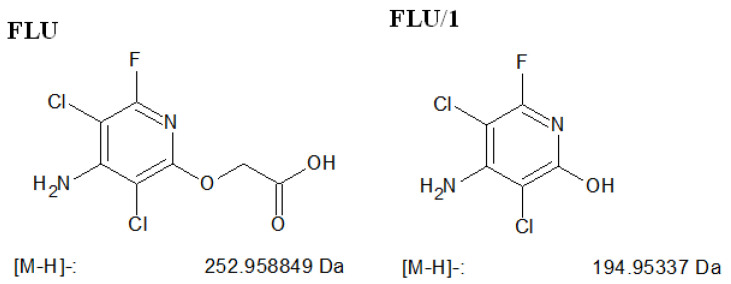
The structure of FLU, and the suggested structure and calculated mass of the formed ion of their detected stable product.

In the case of FLU, the measurements were also performed in negative ion mode. FLU (*m*/*z* = 253.1) and only one stable product, FLU/1 (*m*/*z* = 195.0) was identified after 60 min of irradiation (Figure 18). FLU/1 formed from the decarboxylation of FLU, followed by the formation of a hydroxyl group. This was also reported by Aramendía et al. [55], who mainly attributed the transformation of FLU to reactions with hydroxyl radicals.

## 4. Conclusions

In this paper, we reported the preparation of TiO_2_, ZnO, and MgO nanoparticles using sol–gel method and structural and morphological properties of these powders were investigated by XRD, FTIR, UV/Vis, BET and SEM/EDX techniques. A comparative study was made of the activity of all mentioned nanopowders toward the photocatalytic degradation of two antibiotics (CIP and CEF) and two herbicides (TEM and FLU). TiO_2_ was proved as the most efficient nanopowder using UV and SS. Generally, the addition of lower concentrations of (NH_4_)_2_S_2_O_8_ leads to the faster removal of both antibiotics and herbicide FLU in the presence of TiO_2_. In the case of herbicide TEM, system TiO_2_/(NH_4_)_2_S_2_O_8_ (0.5 mM) under UV light and TiO_2_/(NH_4_)_2_S_2_O_8_ (0.125 mM) under SS were identified as the most efficient and were chosen for further investigations. On the basis of the toxicity, the exposure of wheat seeds to the mixtures of each pollutant and formed intermediates only during germination (imbibition) allowed assessing their effects on further growth of young plants. Finally, in the case of CIP, TEM and FLU, several degradation intermediates were formed and identified by the LC–ESI–MS technique. It is implied that the transformations of herbicides and their intermediates can be mainly attributed to hydroxyl radicals.

## Figures and Tables

**Table 1:** Major properties of investigated emerging pollutants.

Pollutant	Property			
	Group	Molecular Formula	Molecular Weight (g/mol)	p*K*_a_
CIP	Fluoroquinolone antibiotic	C_17_H_18_FN_3_O_3_	331.34	p*K*_a1_ = 6.09 ^a^ p*K*_a2_ = 8.74 ^a^
CEF	Cephalosporin antibiotic	C_18_H_16_N_8_Na_2_O_7_S_3_·3.5H_2_O	661.60	p*K*_a1_ ~ 3.0 ^b^ p*K*_a2_ = 3.2 ^b^ p*K*_a3_ = 4.1 ^b^
TEM	Triketone herbicide	C_17_H_16_ClF_3_O_6_S	440.82	3.18 (20 °C) ^c^
FLU	Synthetic auxins	C_7_H_5_Cl_2_FN_2_O_3_	255.03	p*K*_a1_ = 3.5 ^d^ p*K*_a2_ = 10.9 ^d^

Data extracted from ref. ^a^ [28]; ^b^ [29]; ^c^ [16]; ^d^ [30].

**Table 2:** Surface area, pore size distribution and total pore volume calculated from adsorption–desorption isotherms for TiO_2_, ZnO and MgO materials.

Sample	Surface Area (m^2^/g)	Pore Size Distribution BJH Ads (nm)	Pore Size Distribution BJH Des (nm)	Total Pore Volume (cm^3^/g)
TiO_2_	112	4.293	3.748	0.11980
ZnO	18	3.078	3.711	0.04179
MgO	75	16.826	9.720	0.33360
